# Thusin, a Novel Two-Component Lantibiotic with Potent Antimicrobial Activity against Several Gram-Positive Pathogens

**DOI:** 10.3389/fmicb.2016.01115

**Published:** 2016-07-19

**Authors:** Bingyue Xin, Jinshui Zheng, Hualin Liu, Junhua Li, Lifang Ruan, Donghai Peng, Muhammad Sajid, Ming Sun

**Affiliations:** State Key Laboratory of Agricultural Microbiology, College of Life Science and Technology, Huazhong Agricultural UniversityWuhan, China

**Keywords:** two-peptide lantibiotics, *Bacillus thuringiensis* BGSC 4BT1, thusin, vancomycin, Gram-positive pathogens

## Abstract

Due to the rapidly increasing prevalence of multidrug-resistant bacterial strains, the need for new antimicrobial drugs to treat infections has become urgent. Bacteriocins, which are antimicrobial peptides of bacterial origin, are considered potential alternatives to conventional antibiotics and have attracted widespread attention in recent years. Among these bacteriocins, lantibiotics, especially two-component lantibiotics, exhibit potent antimicrobial activity against some clinically relevant Gram-positive pathogens and have potential applications in the pharmaceutical industry. In this study, we characterized a novel two-component lantibiotic termed thusin that consists of Thsα, Thsβ, and Thsβ' (mutation of Thsβ, A14G) and that was isolated from a *B. thuringiensis* strain BGSC 4BT1. Thsα and Thsβ (or Thsβ') exhibit optimal antimicrobial activity at a 1:1 ratio and act sequentially to affect target cells, and they are all highly thermostable (100°C for 30 min) and pH tolerant (pH 2.0 to 9.0). Thusin shows remarkable efficacy against all tested Gram-positive bacteria and greater activities than two known lantibiotics thuricin 4A-4 and ticin A4, and one antibiotic vancomycin against various bacterial pathogens (*Bacillus cereus, Listeria monocytogenes, Staphylococcus aureus* (MRSA), *Staphylococcus sciuri, Enterococcus faecalis*, and *Streptococcus pneumoniae*). Moreover, thusin is also able to inhibit the outgrowth of *B. cereus* spores. The potent antimicrobial activity of thusin against some Gram-positive pathogens indicates that it has potential for the development of new drugs.

## Introduction

The introduction of antibiotics for the treatment of infections is the most significant scientific achievement of the twentieth century with regard to reducing human morbidity and mortality (Andersson and Hughes, 2010; Brown and Wright, 2016). Unfortunately, the extensive use of antibiotics has resulted in the development of multidrug-resistant pathogens, and mining new antimicrobials is considered to be an effective solution to this problem (Cotter et al., 2013). To date, the investigated alternatives to traditional antibiotics include plant-derived compounds, bacteriophages, RNA-based therapeutics, probiotics, and antimicrobial peptides of different origins (Shanahan, 2010; Burrowes et al., 2011; Kole et al., 2012; Li et al., 2012; Savoia, 2012). Antimicrobial peptides of bacterial origin, known as bacteriocins, are ribosomally synthesized peptides that exert a bactericidal or bacteriostatic effect on other bacteria either of the same species (narrow spectrum) or across genera (broad spectrum) and have been regarded as a promising source of antimicrobials (Cotter et al., 2005, 2013). They include posttranslationally modified bacteriocins and unmodified or cyclic bacteriocins (Cotter et al., 2013).

Lanthipeptides (lantibiotics) are small peptides that undergo extensive posttranslational modification and are considered the best-characterized bacteriocins (Schnell et al., 1988; Willey and Van der Donk, 2007). The posttranslational modifications include the dehydration of Ser and Thr residues to yield dehydroalanine (Dha) and dehydrobutyrine (Dhb) residues, respectively. This dehydration is followed by the stereospecific intramolecular addition of a Cys residue onto the dehydroamino acids to form a lanthionine (Lan) or methyllanthionine (MeLan) bridge. Many lantibiotics exhibit broad-spectrum antimicrobial activity against clinically relevant Gram-positive pathogens, including multidrug-resistant pathogens, and are a promising class of new antibacterial agents (Severina et al., 1998; Cotter et al., 2005, 2013; Dischinger et al., 2014; Sandiford, 2014).

A special subclass of two-component lantibiotics consists of two peptide systems that are formed from two precursor peptides posttranslationally modified to form two distinct active products (Garneau et al., 2002). The individual peptides have little or no antimicrobial activity; however, equimolar concentrations of the two peptides act in synergy to exhibit significantly higher activity (Morgan et al., 2005). At present, a number of two-component lantibiotics have been described, some of which may have applications in the pharmaceutical and food industries (Navaratna et al., 1998; Ryan et al., 1999; Holo et al., 2001; Hyink et al., 2005; Yonezawa and Kuramitsu, 2005; McClerren et al., 2006; Begley et al., 2009; Sawa et al., 2012). For example, the best-studied two-component lantibiotic, lacticin 3147, consists of Ltnα and Ltnβ and exhibits potent antimicrobial activity against a number of pathogenic Gram-positive bacteria, such as methicillin-resistant *Staphylococcus aureus* (MRSA), vancomycin-resistant *Enterococcus faecalis* (VRE), penicillin-resistant *Pneumococcus* (PRP), *Propionibacterium acnes, Streptococcus mutans, Clostridium difficile*, and *Mycobacteria* (Galvin et al., 1999; Morgan et al., 2005; Rea et al., 2007; Carroll et al., 2010; Dobson et al., 2011; Piper et al., 2012). Lacticin 3147 also substantially attenuated *Strep. mutans* biofilm formation (Dobson et al., 2011) and prevented the systemic spread of *Staph. aureus* in mice (Piper et al., 2012). Although there are a number of two-component lantibiotics have been described, the properties of them are different, such as the antimicrobial activity and stability (Willey and Van der Donk, 2007; Knerr and Van der Donk, 2012). Therefore, it's necessary to mine more antimicrobials of that type to satisfy people's demands.

In our previous research, we demonstrated that the *B. cereus* group is an excellent reservoir of novel lanthipeptides, and four types of two-component lanthipeptide gene clusters were predicted in the strains of this group (Xin et al., 2015b). In this study, we identified and characterized one type of the two-component lantibiotics, the thusin gene cluster from a *B. thuringiensis* strain BGSC 4BT1. The two components, Thsα and Thsβ (or Thsβ'), acted synergistically against most of the tested Gram-positive bacteria and were able to inhibit the outgrowth of *Bacillus cereus* spores. In addition, comparison of the antimicrobial activities of thusin, thuricin 4A-4, ticin A4, and vancomycin against six species of Gram-positive bacterial pathogens suggested that thusin may be a promising therapeutic agent.

## Materials and methods

### Strains and culture conditions

The *B. thuringiensis* strain BGSC 4BT1 was kindly provided by the *Bacillus* Genetic Stock Center (BGSC) and propagated on Luria-Bertani (LB) agar plates at 28°C. The other strains, which served as indicator strains, are listed in Table 1.

**Table 1:** **The antimicrobial activity of Thsα, Thsβ, and Thusin**.

**Indicator strain^a^**	**MIC (μM)^b^**		
	**Thsα**	**Thsβ**	**Thusin**
**GRAM-NEGATIVE BACTERIA**			
*Sphingobacterium* Pri1	–	–	–
*Pseudomonas putida* Pri3	–	–	–
*Pseudomonas psychrophila* Pri5	–	–	–
*Escherichia coli* OP50 (Xin et al., 2015a)	–	–	–
*E. coli* BL21 (Xin et al., 2015a)	–	–	–
*Erwinia herbicola* LS005(Xin et al., 2015a)	–	–	–
*Klebsiella pneumoniae* CMCC 46117	–	–	–
*Salmonella paratyphi* CMCC 50093	–	–	–
*Salmonella paratyphi* CMCC 50094	–	–	–
*Shigella dysenteriae* CMCC 51105	–	–	–
*Pseudomonas aeruginosa* ATCC 27853	–	–	–
**GRAM-POSITIVE BACTERIA**			
*Bacillus cereus* ATCC 14579	6.25	12.5	0.78
*Bacillus thuringiensis* BMB171 (Xin et al., 2015a)	6.25	6.25	0.39
*Bacillus pumilus* SCG I (Xin et al., 2015a)	6.25	12,5	0.39
*Bacillus subtilis* Bsn5 (Deng et al., 2011)	6.25	12.5	0.78
*Bacillus amyloliquefaciens* X1 (Xin et al., 2015a)	12.5	12.5	1.56
*Listeria monocytogenes* LM201 (Wu et al., 2015)	6.25	12.5	0.78
*Listeria monocytogenes* LM605	12.5	12.5	0.78
*Staphylococcus aureu*s CMCC 26003	25.0	25	1.56
*Staphylococcus aureu*s ATCC 43300	12.5	25.0	1.56
*Staphylococcus aureu*s MRSA	25.0	25.0	1.56
*Staphylococcus sciuri* Bom1	12.5	12.5	0.78
*Enterococcus faecalis* ATCC 29212	50.0	50.0	3.13
*Streptococcus pneumoniae* ATCC 49619	6.25	12.5	1.56

a *ATCC, American Type Culture Collection. CMCC, China Medical Culture Collection. See references for the source of the marked strains. Unmarked strains were isolated by our group*.

b *The highest concentrations of Thsα, Thsβ, and Thusin were all 100 μM. The minus sign denotes no activity against the indicator strains, even at the highest concentrations of the indicated peptides*.

### The prediction of the lanthipeptide biosynthetic gene cluster and analysis of the promoter and terminator in the thusin gene cluster

The bacteriocin biosynthetic gene clusters were predicted using BAGEL 3.0 (Van Heel et al., 2013). The putative promoter and terminator in the thusin gene cluster were detected using the Softberry BPROM and Find Term software programs, respectively.

### Purification of antibacterial peptides

*B. thuringiensis* BGSC 4BT1 was grown in Luria-Bertani (LB) broth at 28°C. At the early stationary phase (OD_600_≈3.0), the cells were removed by centrifugation at 12,000 rpm for 10 min. The concentration of active substances was measured using Amberlite XAD-7HP (Sigma, St Louis, MO, USA) as described previously (Xin et al., 2015a). Briefly, the cell-free supernatant fluid (5 L) was shaken with 500 g of Amberlite XAD-7HP (Sigma, St. Louis, MO, USA) for 12 h at 4°C. The resin was sequentially washed with 2 L of distilled water and 1 L of 30% (vol/vol) ethanol. The active substances were eluted with 500 mL of 80% (vol/vol) ethanol, pH 2.0, and the eluate was collected and lyophilized into a powder. The generated powder was dissolved in 5 mL of acetonitrile 50% (vol/vol) followed by centrifugation. The resulting supernatant is referred to as antimicrobial crude extract (CE). The CE was analyzed using the Waters 1525 Breeze system. The solvents were (A) HPLC-grade water with 0.1% trifluoroacetic acid (TFA) and (B) acetonitrile, and these solvents were applied using the following gradient: 20–60% B from 0 to 30 min at a flow rate of 1.0 mL/min. The resulting quantity of Thsα, Thsβ, and Thsβ' was quantified by weighing and the compounds were reconstituted in distilled water (0.5 mg/mL).

Vancomycin hydrochloride was purchased from Sigma with purity greater than 90% and was reconstituted in distilled water (0.1 mM). Purification of thuricin 4A-4 and ticin A4 was carried out as described previously (Xin et al., 2015a,b). The purities of thuricin 4A-4, ticin A4, Thsα, Thsβ, and Thsβ' were determined by HPLC using the above described procedure, and the values all exceeded 90%.

### The optimal peptide ratio for the antimicrobial effect of thusin

The activated *B. thuringiensis* BMB171 cultures were subcultured in 5 mL of LB medium (5 × 10^5^ cfu/mL) that contained varying amounts of Thsα and Thsβ (or Thsβ') at 28°C with agitation at 220 rpm for 5 h. Bacterial growth was evaluated by measuring the culture OD_600_.

### Antimicrobial activity assay and determination of minimal inhibitory concentration (MIC) values

The antimicrobial activity of the fermental supernatant of strain BGSC 4BT1 and the eluate in HPLC analysis were assessed using the agar well diffusion method as described previously (Xin et al., 2015b). The minimal inhibitory concentration (MIC) was assessed using the DIN-58940-8 microdilution method (Assadian et al., 2011). Briefly, the test strains were cultivated overnight and diluted to reach 5 × 10^5^ cfu/mL. Tests were performed using 96-well microtiter plates. Each well was filled with 100 μL of serial two-fold dilutions of the antimicrobials and 100 μL of the inoculum and incubated at 37°C for 24 h. All experiments were performed in triplicate. The MIC was defined as the lowest concentration of samples that could inhibit visible growth of the tested strains.

### Structural analysis of Thsα, Thsβ, and Thsβ'

LC-MS and LC-MS/MS were used to analyze the structures of Thsα, Thsβ, and Thsβ' using the Agilent 6540 ultra-high-definition (UHD) accurate-mass quadrupole time of flight (Q-TOF) LC-MS system. The MS operating conditions were as follows: capillary voltage, 3500 V; flow rate of drying gas, 9 L/min; nebulizer pressure, 35 lb/in^2^ gauge; and temperature, 350°C. The scanning range of the Q-TOF was *m/z* 100 to 3000. MS/MS analysis was performed on the doubly and tripled charged ions of each peptide. The target ion fragmented by adding a voltage varied from 20 to 80 V.

### Sensitivity of antimicrobial peptides to temperature and pH

To determine the sensitivities of Thsα, Thsβ, and Thsβ' to pH, aliquots of the bacteriocin preparations (5 × MIC) were adjusted to pH 2.0, 3.0, 7.0, 8.0, 9.0, and 10.0 with 1 M NaOH or 1 M HCl solution followed by incubation at 28°C for 2 h, and the residual antimicrobial activity was measured after neutralizing the sample to pH 6.0. For the thermal stability assay, aliquots of the bacteriocin preparations (5 × MIC) were exposed to 80, 100, and 121°C for 30 min and used for an antimicrobial activity assay. *B. thuringiensis* BMB171 was used as the indicator strain in these two experiments.

### Thsα and Thsβ act sequentially to affect the sensitive strain

The *B. thuringiensis* BMB171 cultures (OD_600_≈0.5) were diluted 10-fold, and 200 μL of the diluted culture was added to 1.5 mL Eppendorf tubes that contained Thsα or Thsβ (at concentrations of 0, 10, 30, and 60 nM). The tubes were incubated at 37°C for 30 min prior to centrifugation at 12,000 rpm for 1 min. The supernatants were removed from each tube, and the cell pellets were washed twice with LB broth, then resuspended in 200 μL of LB broth. Cells that had been treated with only Thsα were added to microtiter wells that contained Thsβ, and cells that had been exposed to only Thsβ were added to microtiter wells that contained Thsα (at concentrations of 0, 10, 30, and 60 nM). The microtiter plates were incubated at 28°C and monitored at hourly intervals for 5 h. In addition, cells exposed to Thsα and Thsβ in combination at a ratio of 1:1 served as a control.

### Inhibition of *Bacillus cereus* spore outgrowth

Germination assays were conducted as described previously (Hornstra et al., 2005) with few modifications. *B. cereus* ATCC14579 spores were activated by incubation at 70°C for 15 min and were then transferred into LB broth at an optical density at 600 nm of 0.8. After adding different amounts of antimicrobials, the germination process was followed by monitoring the optical density at 600 nm every 10 min.

### Nucleotide sequence accession number

The whole-genome shotgun sequencing results for *B. thuringiensis* BGSC 4BT1 have been deposited in GenBank under accession no. LILG00000000. The nucleotide sequence of the thusin gene cluster has been deposited in GenBank under accession no. KT454399.

## Results

### Characterization and identification of one two-component lanthipeptide biosynthetic gene cluster in *B. thuringiensis* BGSC 4BT1

**Figure 1:**
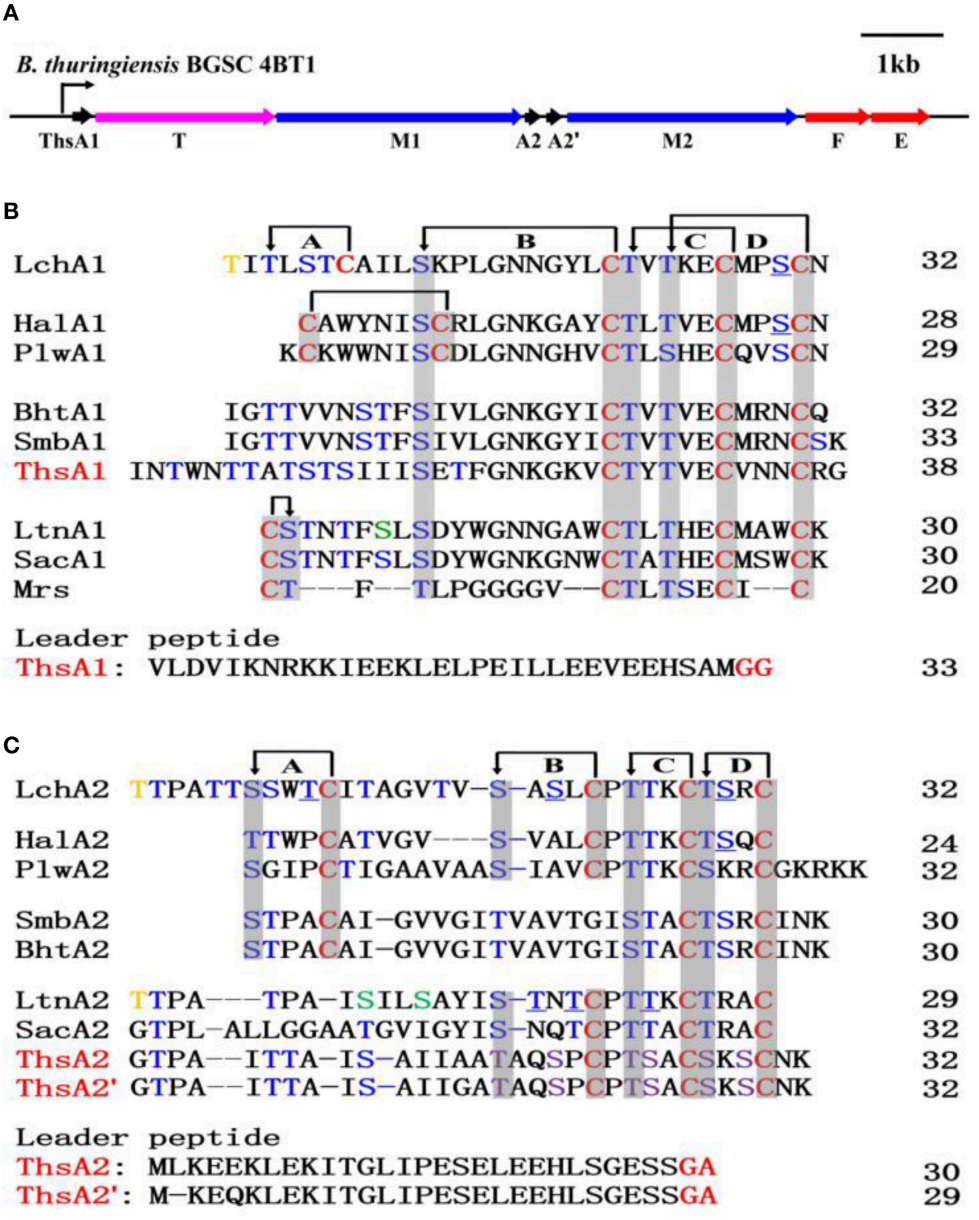
**Thusin biosynthetic gene cluster in ***B. thuringiensis*** BGSC 4BT1 and amino acid sequence alignment of three precursor peptides with class II lantibiotic propeptides. (A)** Line diagram of the thusin gene cluster in *B. thuringiensis* BGSC 4BT1. Bent arrow represents the promoter of the thusin gene cluster. Amino acid sequence alignment of ThsA1 **(B)**, ThsA2 and ThsA2′ **(C)** with other reported class II lantibiotic propeptides. Cys residues are marked in red. Ser/Thr residues that are dehydrated are shown in blue, and non-dehydrated residues are underlined. Ser/Thr residues, post-translationally modified to D-Ala/Obu, are shown in green/yellow. Thioether and disulfide bonds are marked with arrows and are boxed in gray. Two of the six Ser/Thr residues are not dehydrated in the mature peptides of ThsA2 and ThsA2′, but these residues could not be conclusively identified based on the current data; these six amino acids are displayed in violet. Thioether bridging rings in the Lchα and Lchβ peptides are indicated with the capital letters A, B, C, and D. The probable protease cleavage site sequences (GG) are shown in red.

Our previous research demonstrated that *B. cereus* group strains are a prolific source of novel lantibiotics, and four types of the two-component lanthipeptide biosynthetic gene clusters that have not yet been biochemically characterized were predicted in the strains of this group (Xin et al., 2015b). Among these clusters, one type of putative two-component lanthipeptide gene cluster can be mined from the genome sequences of *B. thuringiensis* BGSC 4BD1 and 4BT1, and we functionally verified this gene cluster in strain 4BT1. This gene cluster, ~10 kb in length, consists of eight genes, including three structural genes (*thsA1, thsA2*, and *thsA2*′), two genes encoding a posttranslational modification enzyme (*thsM1 and thsM2*), two genes encoding an ABC transporter that could be involved in immunity (*thsEF*), and one gene (*thsT*) encoding a transporter (Figure 1A). Only one putative promoter was predicted in the upstream *thsA1* gene, and no terminator was found in the DNA region of this gene cluster (Figure 1A). In addition, three putative precursor peptides are characterized by an N-terminal leader sequence with a conserved G (G/A) cleavage site (Figures 1B,C). The amino acid sequence of ThsA1 (71 aa) shows the highest identity with LchA1 (43.8%). (Shenkarev et al., 2010) The sequences of ThsA2 (62 aa) and ThsA2' (61 aa) are nearly identical (95% identity), and they are most similar to that of LtnA2 (33.3 and 30.8%, respectively) (Ryan et al., 1999).

### Purification and identification of Thsα, Thsβ, and Thsβ'

**Figure 2:**
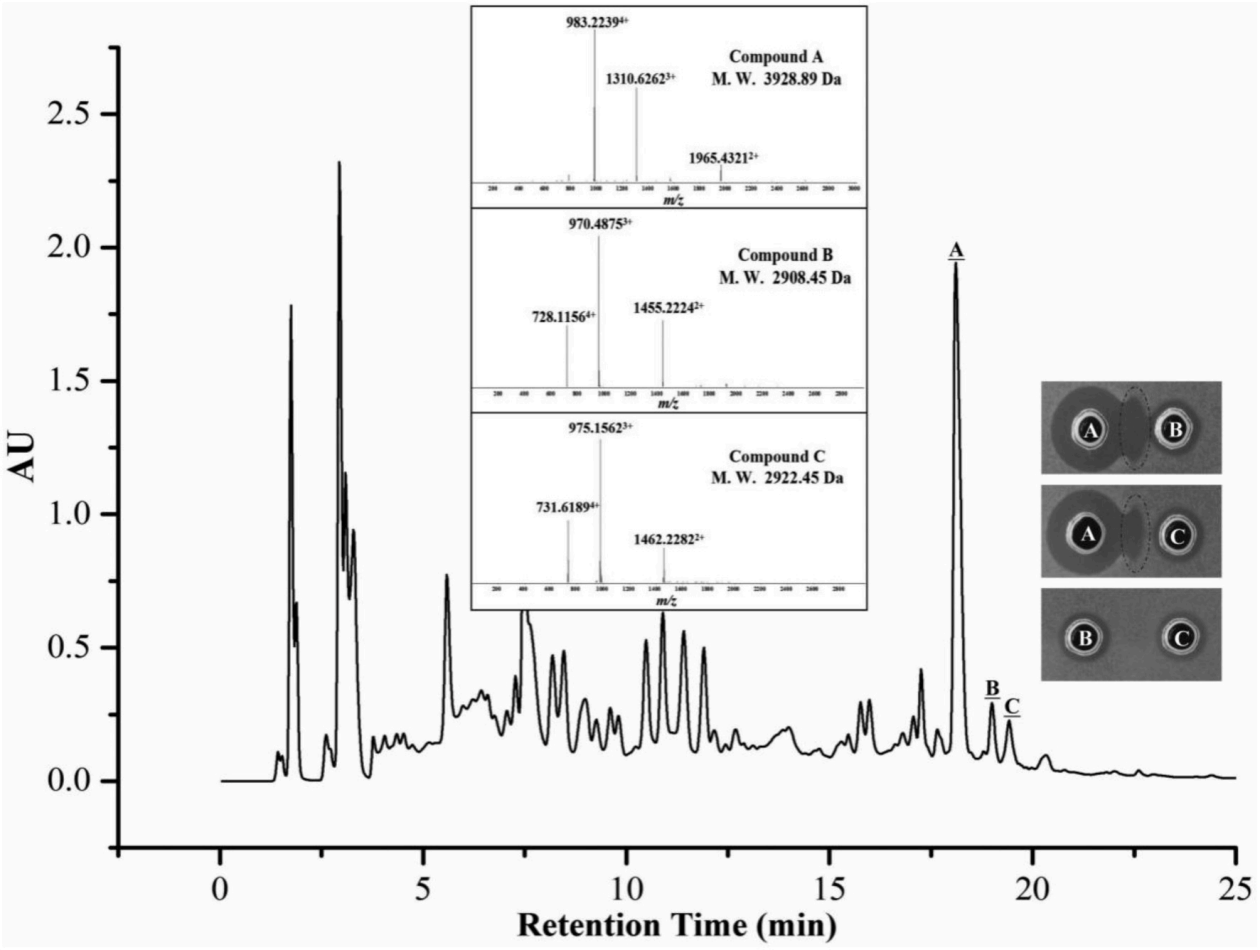
**LC-MS analysis of a crude extract from a ***B. thuringiensis*** BGSC 4BT1 culture supernatant**. Only three fractions (A, B, and C) corresponding to three peaks with retention times of 18.1, 19.0, and 19.4 min were active against *B. thuringiensis* BMB171. The complementary activity was observed between fractions A and B as well as between fractions A and C but not between fractions B and C.

The kinetics of the antimicrobial substance production assay demonstrated that strain 4BT1 produced antimicrobials during the exponential phase (Figure S1). The antimicrobials from a 12 h culture of 4BT1 in LB medium (the exponential phase of growth) were concentrated on Amberlite XAD-7 HP resin and separated by reverse-phase HPLC (RP-HPLC). As shown in Figure 2, only three fractions corresponding to three peaks with retention times of 18.1 min, 19.0 min and 19.4 min were active against *B. thuringiensis* BMB171. Note that complementary activity was observed between fractions A and B as well as between fractions A and C but not between fractions B and C. This phenomenon was similar to that observed for a previously reported two-component lantibiotic, lacticin 3147 (Ryan et al., 1999). Moreover, the LC-MS data showed that the molecular mass of fraction A was 3928.89 Da (Figure 2). The calculated molecular mass of the predicted mature peptide of ThsA1, Thsα, was 198.74 Da higher than the measured mass, indicating that all 11 serine and threonine residues were dehydrated. Two compounds, B and C, with molecular masses of 2908.45 Da and 2922.45 Da, were detected in fractions B and C, respectively (Figure 2). The calculated molecular masses of the predicted products of the structural genes *thsA2* and *thsA2*′ were 3065.52 Da and 3051.50 Da, respectively, which were each 143.07 Da higher than the measured masses of compounds C and B, indicating that eight of the ten serine or threonine residues were dehydrated.

**Figure 3:**
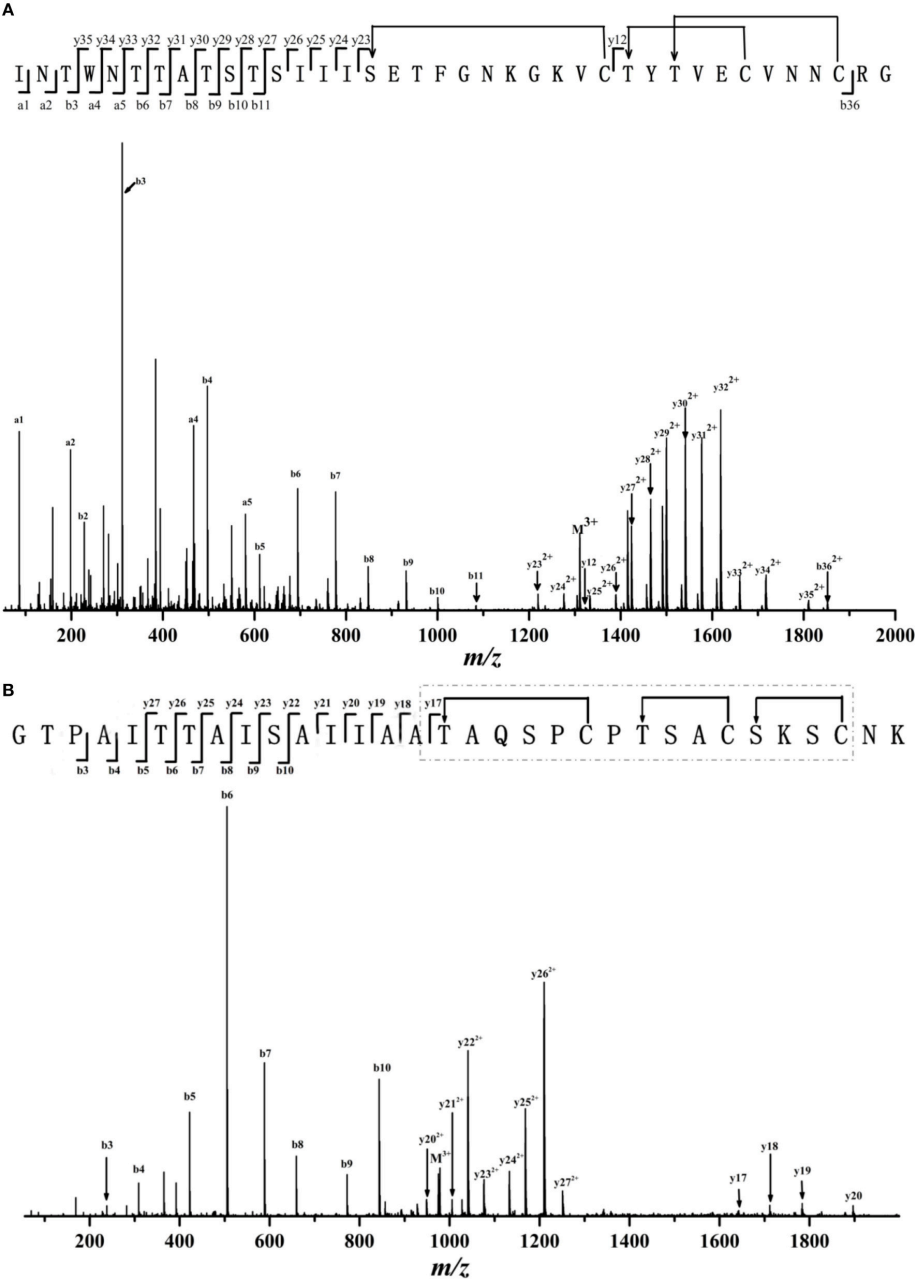
**ESI-MS/MS spectra and proposed primary structure of Thsα (A) and Thsβ (B)**. Fragment ions are indicated. The putative thioether bridging rings of Thsα and Thsβ are shown. Two of the six Ser/Thr residues are not dehydrated in mature peptides of ThsA2, but these residues could not be conclusively identified based on the current data; these six amino acids are shown in gray. The putative thioether bridging rings of Thsβ in the dashed boxes were presumed based on the reported class II lantibiotics which had been identified their structures.

We then used LC-MS/MS to analyze the detailed sequence information of Thsα, Thsβ, and Thsβ'. As shown in Figure 3A and Table S1, all marked fragment ions of compound A corresponded to fragments of Thsα. No cleavage was observed from Ser16 to Cys26 or from Thr27 to Cys36, indicating the presence of an intramolecular thioether bridge (Lan and/or MeLan). The marked fragments of compound B correspond to the fragments of Thsβ' (Figure S2 and Table S3), and those of compound C correspond to the fragments of Thsβ (Figure 3B and Table S2). The molecular mass difference of fragment ions b5, b6, and b7 was 83.03 Da, and that of fragment ions y22 and y23 was 71.04 Da, indicating that Thr6, Thr7, and Ser10 were dehydrated (Figure 3B and Figure S2). Four of the six residues (Ser19, Ser24, Ser27, Ser29, Thr16, and Thr23) were also dehydrated, but we could not accurately identify these residues based on the current data. In addition, no cleavage was observed from Thr16 to Cys30 (Figure 3B and Figure S2), suggesting the presence of intramolecular thioether bridges, but we could not determine the precise structure from the current data. Given the MS/MS analysis and the structural similarity (thioether bridging rings) between thusin and the reported two-component lantibiotics (Figures 1B,C), we proposed possible Lan and MeLan bridges in Thsα, Thsβ, and Thsβ' (Figure 3 and Figure S2).

### Thsα and Thsβ function optimally at a 1:1 ratio

**Figure 4:**
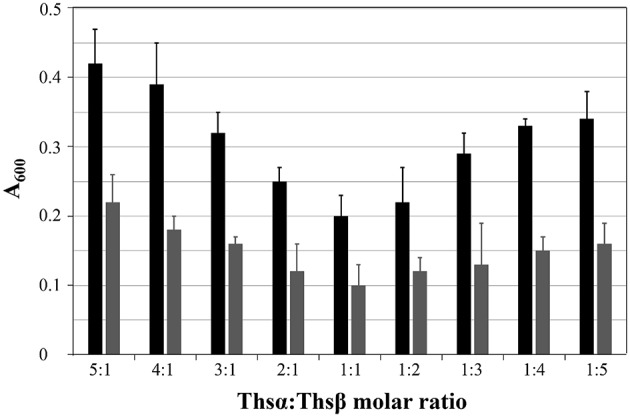
**Effects of the Thsα and Thsβ mixture on ***B. thuringiensis*** BMB171 growth at varying ratios of the peptides, with different total concentrations displayed as gray (60 nM) and black (30 nM) bars**.

Before assessing the specific antimicrobial activity of thusin, the optimal ratio of Thsα to Thsβ (or Thsβ') that maximized the bioactivity was determined. The ability of mixtures containing various amounts of Thsα and Thsβ (or Thsβ') to inhibit the growth of *B. thuringiensis* BMB171 was examined. As shown in Figure 4, thusin is active against the indicator strain *B. thuringiensis* BMB171 at nanomolar levels, and Thsα and Thsβ exhibit maximum activity at a 1:1 ratio. The combination of Thsα and Thsβ' also showed the same result (data not shown). The optimal synergy of Thsα and Thsβ (or Thsβ') at a 1:1 ratio is consistent with reports of other two-component lantibiotics, such as staphylococcin C55, plantaricin W, lacticin 3147, and haloduracin (Navaratna et al., 1998; Holo et al., 2001; Morgan et al., 2005; Oman and Van der Donk, 2009).

### The antimicrobial activity and stability of thusin

**Figure 5:**
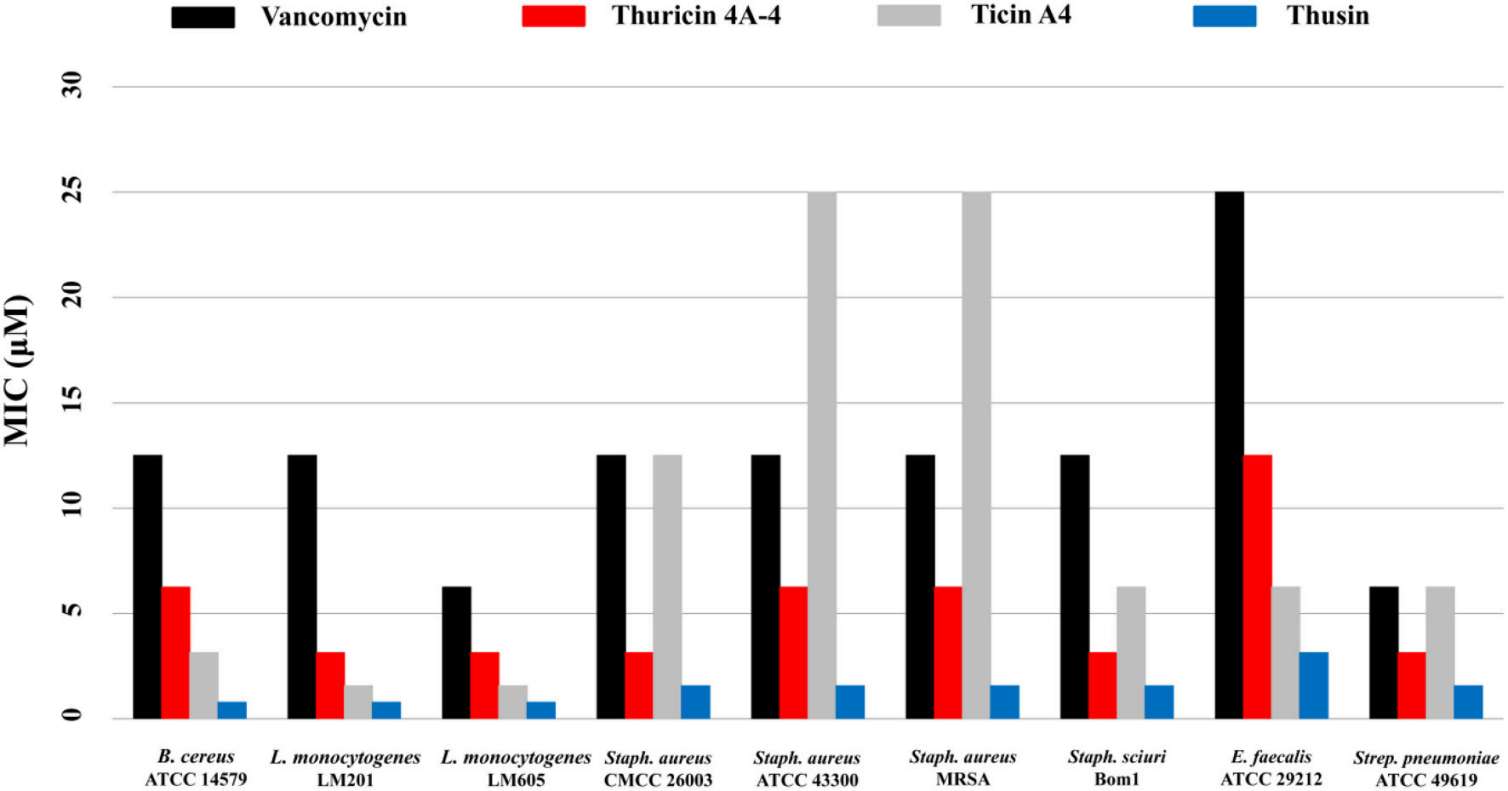
**Comparison of the antimicrobial activities of thusin, ticin A4, thuricin 4A-4, and vancomycin**.

The biological activities of the HPLC-purified Thsα, Thsβ, and Thsβ' against a battery of Gram-positive and Gram-negative bacteria were measured. Thsα, Thsβ, and Thsβ' were active against all tested Gram-positive bacteria, including *B. amyloliquefaciens, B. cereus, B. thuringiensis, B. subtilis, B. pumilus, E. faecalis, L. monocytogenes, Staph. aureus, Staph. Sciuri*, and *Strep. pneumoniae*, but not against any of the tested Gram-negative bacteria (Table 1). The antimicrobial activities of Thsβ and Thsβ' were essentially comparable, so we only showed the data for Thsβ. The Thsα and Thsβ mixture at a molar ratio of 1:1 had a 4- to 16-fold increase in efficacy compared with the peptides used individually. Therefore, Thsα and Thsβ could synergistically inhibit Gram-positive bacteria. In addition, we also compared the antimicrobial activity of thusin, thuricin 4A-4, ticin A4, and vancomycin against six species of Gram-positive pathogens (Figure 5). The MIC determinations revealed that thusin had higher activity against all tested Gram positive bacterial pathogens than those of thuricin 4A-4, ticin A4, and vancomycin. Thusin had 8-fold higher activity against *L. monocytogenes* LM605, *Staph. aureus* CMCC 26003, *Staph. aureus* ATCC 43300, *Staph. aureus* MRSA, *Staph. sciuri* Bom1, and *E. faecalis* ATCC 29212 than vancomycin. Thusin showed 16-fold higher activity against *B. cereus* ATCC 14579 and *L. monocytogenes* LM201 than vancomycin. In addition, thusin displayed 2-fold to 16-fold higher activity against nine tested indicator bacteria than those of thuricin 4A-4 and ticin A4 (Figure 5). These results suggested that thusin might be more effective at treating infections caused by Gram-positive pathogens than thuricin 4A-4, ticin A4 and even vancomycin.

**Figure 6:**
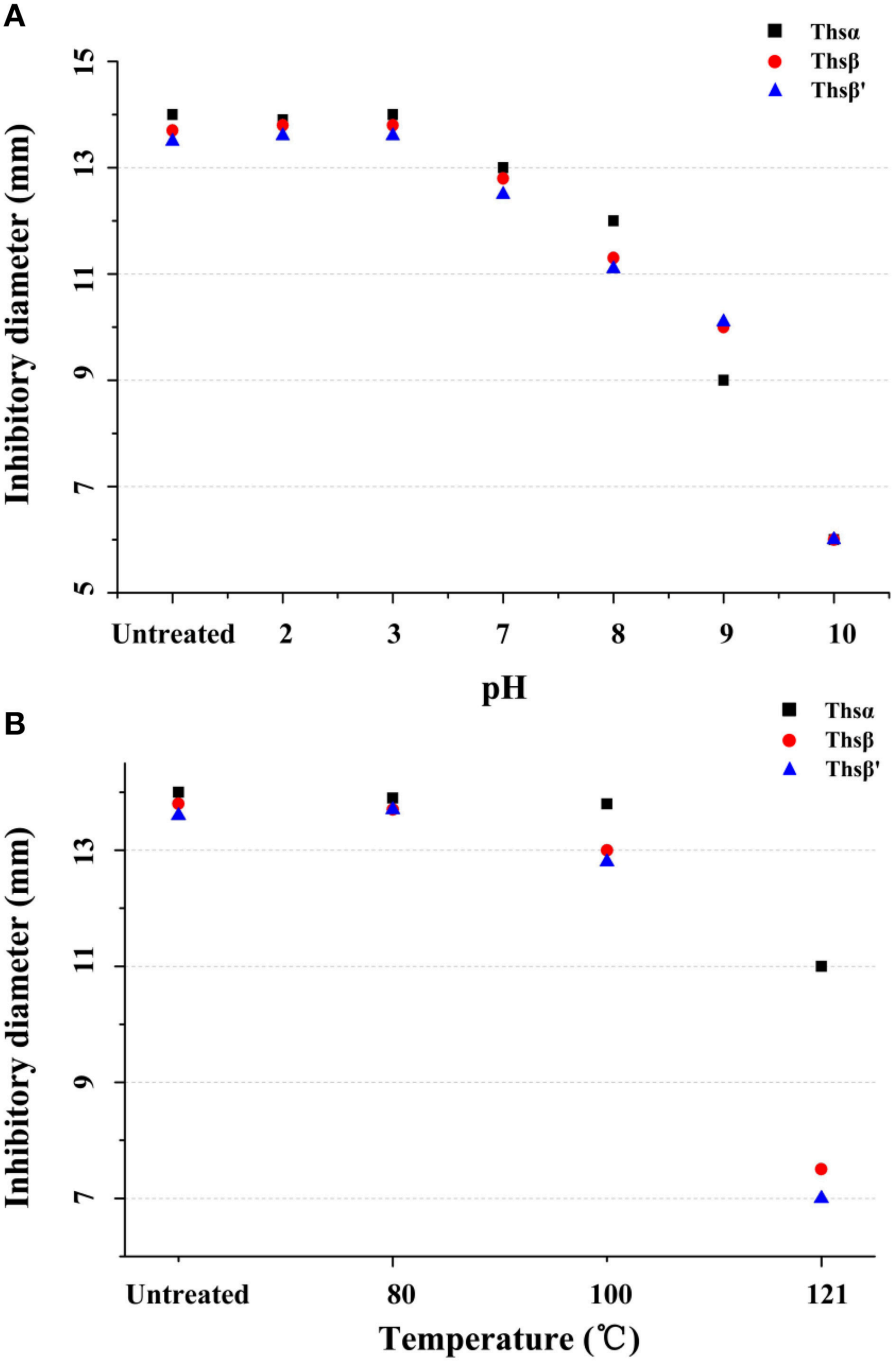
**Thermal and pH stabilities of Thsα, Thsβ, and Thsβ'**. *B. thuringiensis* BMB171 was used as the indicator strain in these two experiments. **(A)** The stabilities of Thsα, Thsβ and Thsβ' under various pH conditions. **(B)** The sensitivities of Thsα, Thsβ, and Thsβ' to temperature. Antimicrobial activity was assessed using the agar well diffusion method. Well size = 6 mm. All assays were repeated at least three times, and representative results are shown.

The sensitivities of Thsα, Thsβ, and Thsβ' to pH and temperature were also tested. As shown in Figure 6A, Thsα, Thsβ, and Thsβ' were all stable under acidic conditions, but were not stable under neutral and alkaline conditions. All of the peptides totally lost their activity when they were incubated at pH 10.0 for 2 h. In addition, Thsα, Thsβ, and Thsβ' all remained active after incubation at 80 and 100°C for 30 min (Figure 6B). Thsα retained approximately 50% of its activity when it was autoclaved at 121°C for 30 min, but the residual antibacterial activities of Thsβ and Thsβ' were greatly decreased.

### Thsα and Thsβ act sequentially to affect sensitive strains

**Figure 7:**
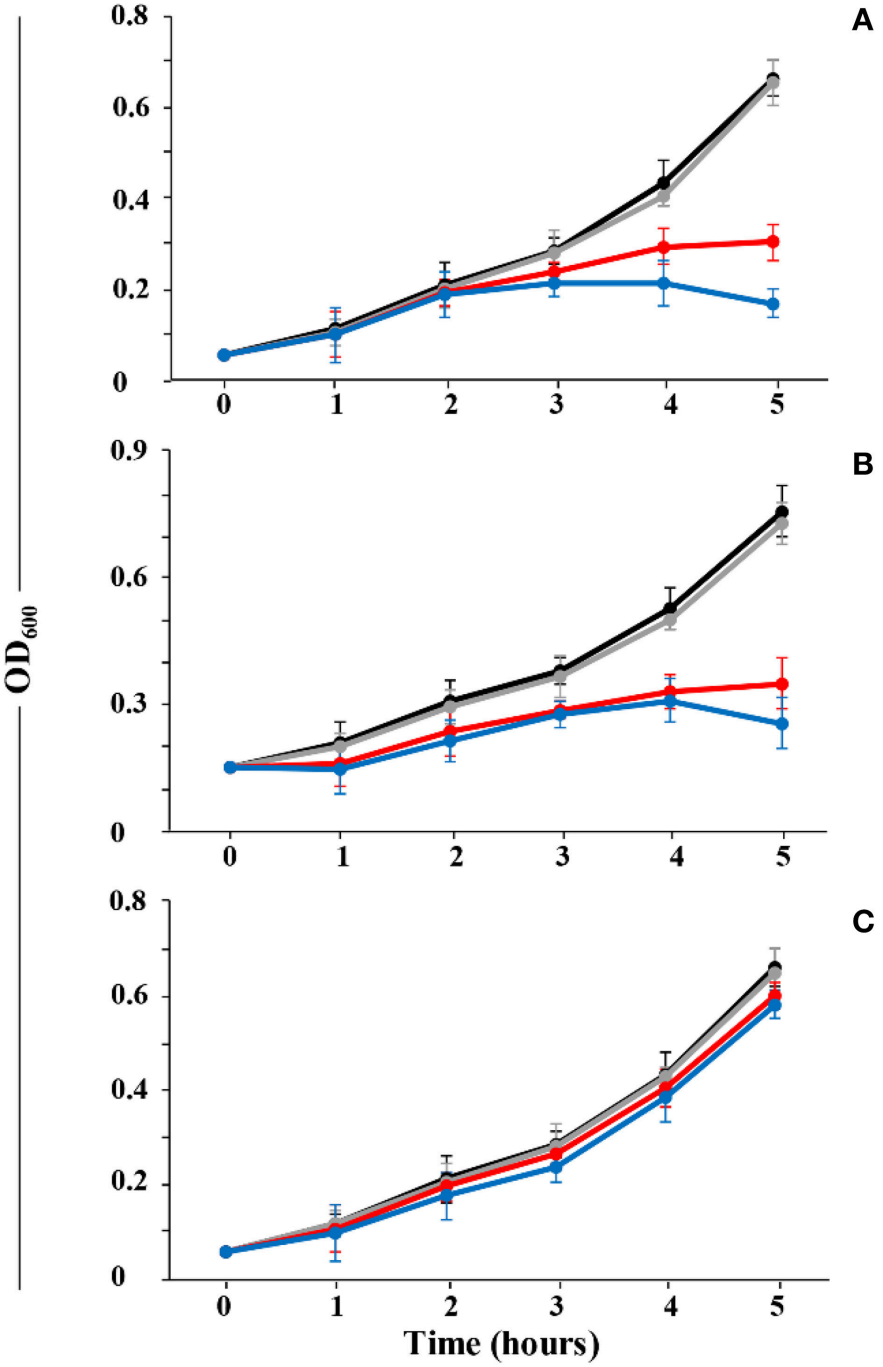
**Sequential activity of thusin peptides against ***B. thuringiensis*** BMB171. (A)** Thsα and Thsβ simultaneously added. **(B)** Thsα followed by Thsβ addition. **(C)** Thsβ followed by Thsα addition. Different concentrations are displayed as black (0 nM), gray (10 nM), red (30 nM), and blue (60 nM) lines. The means and standard deviations are presented.

To reveal the potential roles of the individual peptides of thusin, the peptides were sequentially added and cell growth was examined. As indicated in Figure 7B, when the sensitive strains were incubated with Thsα prior to incubation with Thsβ, cell growth was inhibited. When the cells were first exposed to Thsβ and were subsequently treated with Thsα, growth inhibition was not observed (Figure 7C). When Thsα and Thsβ peptides were added in combination, growth inhibition was observed (Figure 7A).

### Thusin can inhibit the outgrowth of *Bacillus cereus* spores

**Figure 8:**
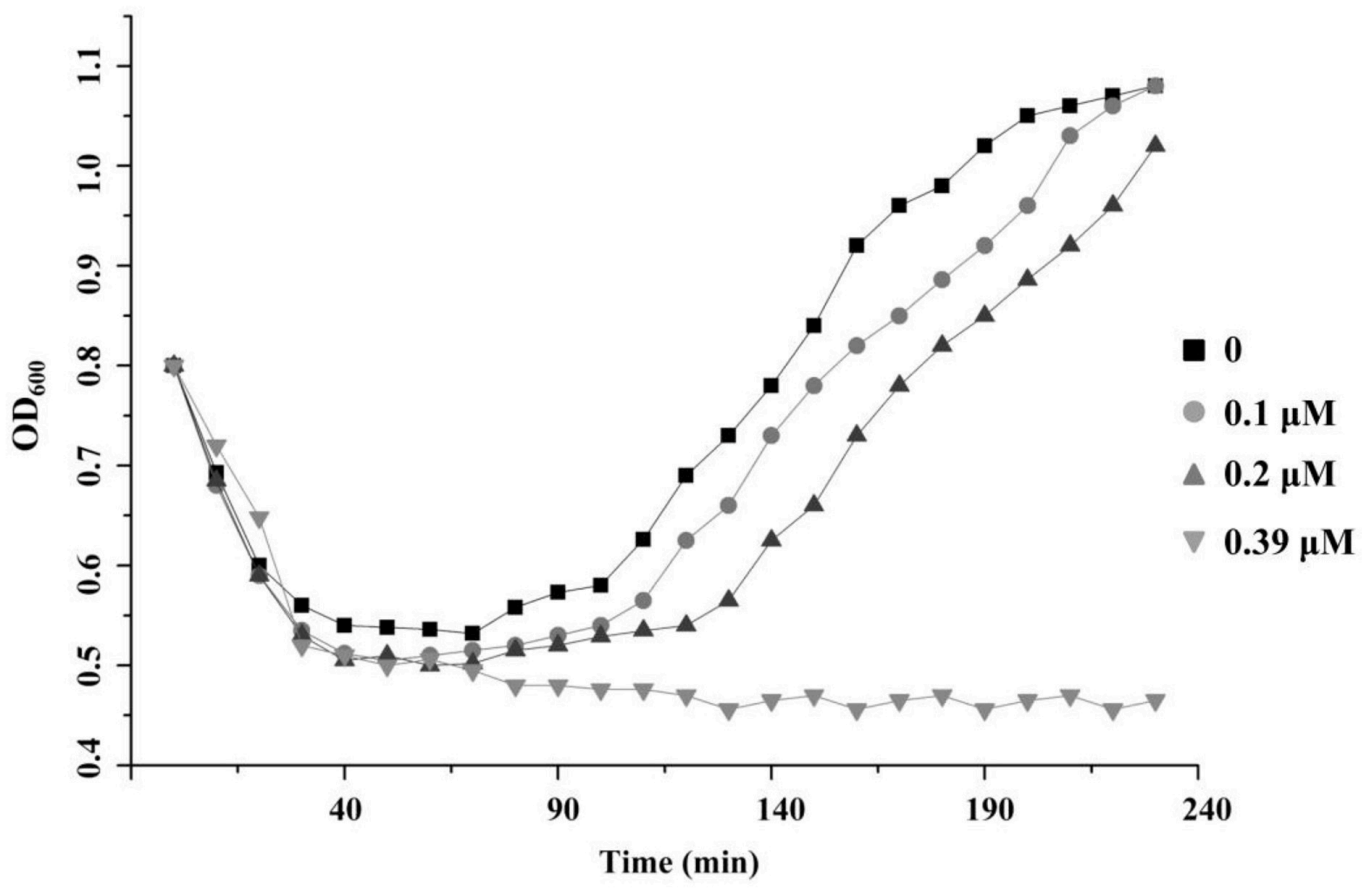
**Inhibition of ***Bacillus cereus*** spore outgrowth by thusin**. The germination process of *B. cereus* ATCC14579 spores was monitored by measuring the optical density at 600 nm every 10 min.

The ability of thusin to prevent spore outgrowth was assessed. The optical density of the samples at 600 nm indicated that thusin could inhibit the outgrowth of *B. cereus* spores when the concentration reached 0.39 μM (Figure 8). In addition, Thsα, Thsβ and Thsβ' could also inhibit the outgrowth of spores when the concentration was greater than 6.25 μM, 12.5 μM and 12.5 μM, respectively (data not shown). Therefore, Thsα and Thsβ (or Thsβ') could synergistically inhibit the outgrowth of *B. cereus* spores.

## Discussion

The increasing incidence of drug-resistant pathogens has prompted the pharmaceutical and scientific communities to investigate alternatives to traditional antibiotics, such as bacteriocins. Bacteriocins exhibit several desirable properties that make them suitable as alternatives to traditional antibiotics: (i) they are potent (in vitro and in vivo), (ii) they are minimally toxic, (iii) they can be produced in situ by probiotics, and (iv) they are amenable to bioengineering (Cotter et al., 2013). A subgroup of bacteriocins, lantibiotics, especially two-component lantibiotics, exhibit potent antimicrobial activity against clinically relevant pathogens, including multidrug-resistant strains, and they are being extensively researched as a potential new source of antimicrobials to treat infections (Cotter et al., 2013; Dischinger et al., 2014; Sandiford, 2014). Given the high potency and potential clinical applications of two-component lantibiotics, this study aimed to mine novel two-component lantibiotics that exhibit excellent properties, especially in terms of antibacterial activity against bacterial pathogens in the *B. cereus* group. Finally, we identified and characterized one type of novel two-component lantibiotic, the thusin gene cluster in *B. thuringiensis* strain BGSC 4BT1. The mature peptides, Thsα and Thsβ (or Thsβ'), act synergistically to potently inhibit several Gram-positive pathogens, including *B. cereus, L. monocytogenes, Staph. aureus* (MRSA), *Staph. sciuri, E. faecalis*, and *Strep. pneumoniae*. To our knowledge, thusin is the first reported two-component lantibiotic in the *B. cereus* group and has potential applications in the pharmaceutical industry.

The optimal ratio of Thsα to Thsβ (or Thsβ') that maximized bioactivity was 1:1. Due to the differences in the maximum absorption wavelengths of Thsα and Thsβ, the absorption peak areas of Thsα, Thsβ and Thsβ' at 220 nm differed (Figure 2), but the number of moles of the compounds was ~2:1:1 (data not shown). Although the three structural genes shared one promoter, the transcription of *thsA1* likely exceeded that of *thsA2* and *thsA2*′. Consequently, the yields of Thsα, Thsβ and Thsβ' were close to the optimal ratio to maximize the effect on cells.

In our previous study, we identified two single-component lantibiotics, thurisin 4A-4, and ticin from *B. thuringiensis* T01001 and BMB3201, respectively (Xin et al., 2015a,b). We found that the cell-free supernatants of *B. thuringiensis* T01001, BMB3201, and BGSC 4BT1 exhibited comparable antimicrobial activity against most of the tested Gram-positive strains (data not shown). The production of thurisin 4A-4 and ticin were both ~1 mM/L, but thusin was only 0.1 to 0.2 mM/L. Comparison of antimicrobial activity between thusin, thuricin 4A-4, and ticin A4 showed that thusin had higher activity against all tested indicator bacteria (Figure 5). Therefore, the antimicrobials produced by *B. thuringiensis* BGSC 4BT1 was less than those from two other strains but played a comparable activity. This result indicated that the production of two-component lantibiotics was more effective than the production of single-component lantibiotics based on the survival and adaptability of the producer strains in various ecological environments. Another way, the low yield of thusin will not be conducive to its future application, and we will take some genetic engineering strategies to improve the production of thusin in further research. For example, overexpression of thusin self-protection genes in the producer strain (Heinzmann et al., 2006).

Comparison of antibacterial activity between thusin and vancomycin demonstrated that thusin had a higher activity against all tested Gram-positive pathogens. The glycopeptide antibiotic vancomycin was introduced clinically nearly 60 years ago and has been widely used to treat severe infections due to Gram-positive bacteria resistant to β-lactam antibiotics, in particular the infection caused by methicillin-resistant *S. aureus* (MRSA) (Mainardi et al., 2008). However, the emergence of vancomycin-resistant enterococci (VRE) and vancomycin-resistant staphylococci attenuated its application in recent years (Leclercq et al., 1988; Uttley et al., 1988; Centers for Disease Control and Prevention, 1997; Chang et al., 2003; Courvalin, 2006). In addition, thusin also showed higher activity against all tested indicator bacteria than thuricin A4 and Ticin A4. These results suggested that thusin had obvious advantages in terms of antimicrobial activity, and also indicated the potential for its use as an alternative to conventional antibiotics against the infections caused by Gram-positive pathogens.

Lipid II has been identified as the target molecule of a number of lantibiotics, such as mersacidin. The C ring of mersacidin can bind to lipid II and inhibit transglycosylation in a Ca^2+^-dependent manner, and this structure is conserved in other class II lantibiotics (Brötz et al., 1998; Hsu et al., 2003; Knerr and Van der Donk, 2012), including Thsα. Recent studies have revealed the mode of action of the two-component lantibiotics lacticin 3147 and haloduracin: the α-peptide first interacts specifically with lipid II and the lipid II: α-peptide complex is then able to recruit the β-peptide to form an active three-component complex and subsequently inhibit cell wall biosynthesis to form small pores in the cell membrane (Morgan et al., 2005; Wiedemann et al., 2006; Oman and Van der Donk, 2009). We found that Thsα and Thsβ acted sequentially to affect the sensitive strain *B. thuringiensis* BMB171, and this phenomenon was identical to that observed for the two-component lantibiotics lacticin 3147 and haloduracin. We speculated that Thsα binds the cell wall precursor lipid II, and the lipid II:Thsα complex is required for Thsβ to exert its synergistic effect to inhibit cell wall biosynthesis and form pores in the cell membrane. We will test this hypothesis in future studies.

*B. cereus* is a human pathogen that causes diarrheal or emetic-type illnesses (Ramarao and Sanchis, 2013). Its highly resistant spores can survive in food processing treatments and can be present in final products, where they may lead to food spoilage and food-borne illness (Warda et al., 2015). Previous studies have shown that some lantibiotics are able to inhibit the outgrowth of *Bacillus* spores (Liu and Hansen, 1993; Oman and Van der Donk, 2009; Gut et al., 2011). In this study, we revealed that the components of thusin synergistically prevented the outgrowth of *B. cereus* spores. To date, only the molecular basis by which nisin inhibits spore outgrowth has been extensively studied. Specifically, nisin utilizes lipid II as the germinated spore target during outgrowth inhibition and causes membrane disruption (Liu and Hansen, 1993; Gut et al., 2011). The mechanism by which two-component lantibiotics such as thusin prevent the outgrowth of *B. cereus* spores may differ from that of the single-component lantibiotic nisin; we will investigate this mechanism in future studies. However, the ability of thusin to inhibit the outgrowth of *B. cereus* spores demonstrates its potential application in the food industry.

Finally, we have to emphasize that the systematic safety testing of thusin and its antimicrobial activity *in vivo* are crucial for its successful application as a new drug or food additive. We have determined that thusin (>1 mg/mL) had no hemolytic activity (data not shown), and more detailed testing will be performed to assess the feasibility of its application in further study.

## Author contributions

BX, JZ, and MS designed research; BX, HL, JL, and MSa performed research; BX, JZ, LR, DP, and MS analyzed data; BX and MS wrote the paper.

### Conflict of interest statement

The authors declare that the research was conducted in the absence of any commercial or financial relationships that could be construed as a potential conflict of interest.
