# Supplementary material for: Thusin, a Novel Two-Component Lantibiotic with Potent Antimicrobial Activity against Several Gram-Positive Pathogens
Source: Front Microbiol. 2016 Jul 19;7:1115. doi: 10.3389/fmicb.2016.01115 (PMC4949975; doi:10.3389/fmicb.2016.01115)
Supplement: Supplementary file 1 [file Image1.PDF]

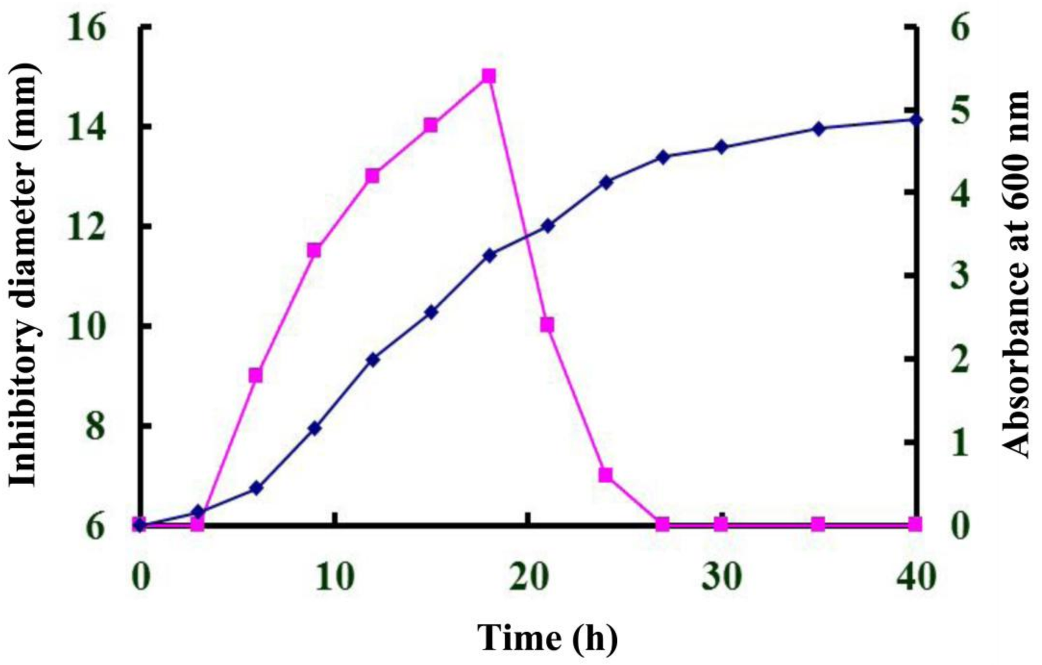

Fig. S1. Kinetics of antimicrobial substances produced during the growth of *B. thuringiensis* BGSC 4BT1.
