# Supplementary material for: Thusin, a Novel Two-Component Lantibiotic with Potent Antimicrobial Activity against Several Gram-Positive Pathogens
Source: Front Microbiol. 2016 Jul 19;7:1115. doi: 10.3389/fmicb.2016.01115 (PMC4949975; doi:10.3389/fmicb.2016.01115)
Supplement: Supplementary file 2 [file Image2.PDF]

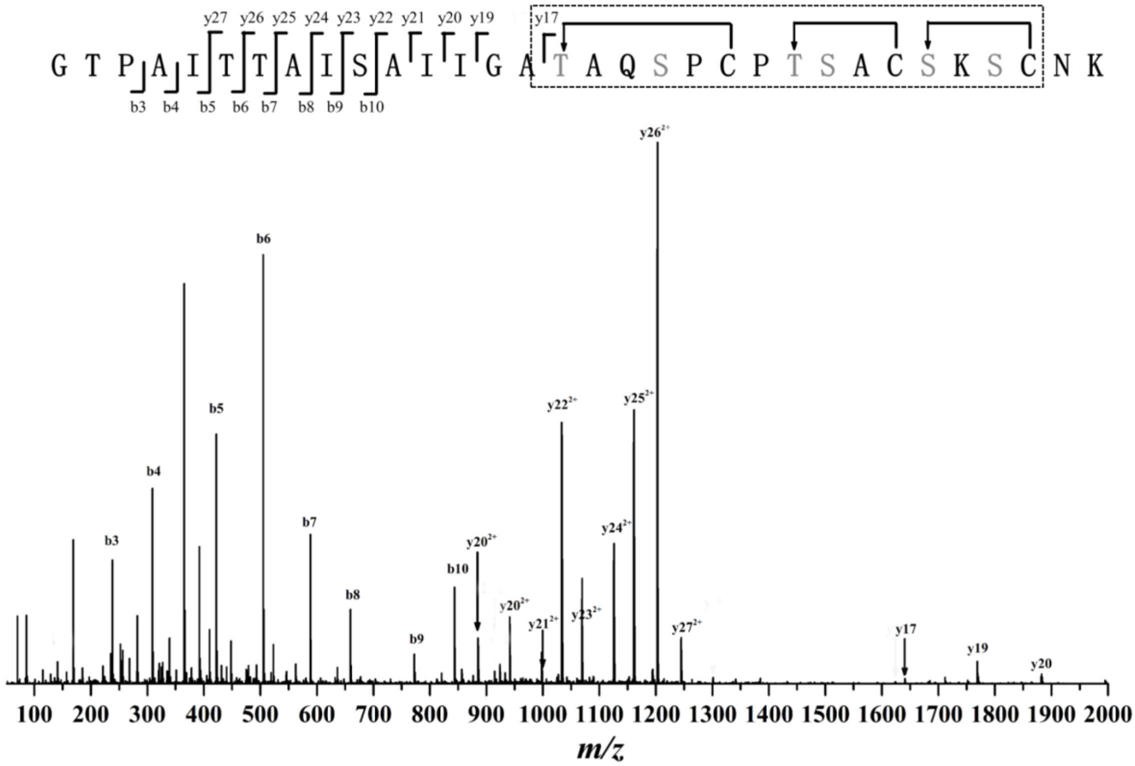

Fig. S2. ESI-MS/MS spectra and proposed primary structure of Thsβ'. Fragment ions are indicated. The putative thioether bridging rings of Thsβ' are shown. Two of six Ser/Thr residues are not dehydrated in mature peptides of ThsA2', and these residues could not be conclusively identified based on the current data; these six amino acids are shown in gray. The putative thioether bridging rings of Thsβ' in the dashed boxes were presumed by the reported class II lantibiotics which had been identified their structures.
