# Supplementary material for: Thusin, a Novel Two-Component Lantibiotic with Potent Antimicrobial Activity against Several Gram-Positive Pathogens
Source: Front Microbiol. 2016 Jul 19;7:1115. doi: 10.3389/fmicb.2016.01115 (PMC4949975; doi:10.3389/fmicb.2016.01115)
Supplement: Supplementary file 3 [file Table1.PDF]

Table S1 Detailed MS/MS data of Thsα

Fragment ion	Measured *m/z*	Fragment ion	Measured *m/z*
a1	86.0967	y12	1322.5052
a2	198.0875	y23	1219.5463^{2+}
b2	228.134	y24	1276.083^{2+}
b3	311.1717	y25	1332.6309^{2+}
a4	467.2039	y26	1389.1763^{2+}
b4	497.2506	y27	1423.6835^{2+}
a5	580.2846	y28	1465.2029^{2+}
b5	611.2946	y29	1499.7140^{2+}
b6	694.3321	y30	1541.2313^{2+}
b7	777.3655	y31	1577.2486^{2+}
b8	848.4027	y32	1618.2659^{2+}
b9	931.4408	y33	1660.2850^{2+}
b10	1000.4582	y34	1717.8150^{2+}
b11	1084.5017	y35	1810.8490^{2+}
b36	1851.8659^{2+}		
