# Supplementary material for: Thusin, a Novel Two-Component Lantibiotic with Potent Antimicrobial Activity against Several Gram-Positive Pathogens
Source: Front Microbiol. 2016 Jul 19;7:1115. doi: 10.3389/fmicb.2016.01115 (PMC4949975; doi:10.3389/fmicb.2016.01115)
Supplement: Supplementary file 4 [file Table2.PDF]

Table S2 Detailed MS/MS data of Thsβ

Fragment ion	Measured *m/z*	Fragment ion	Measured *m/z*
b3	238.1188	y27	1251.1088^{2+}
a4	282.1813	y26	1210.0909^{2+}
b4	309.1562	y25	1168.5742^{2+}
b5	422.2389	y24	1133.0501^{2+}
b6	505.2768	y23	1076.4953^{2+}
b7	588.3143	y22	1040.9954^{2+}
b8	659.3523	y21	1005.0121^{2+}
b9	772.4357	y20	1895.8567, 948.9352^{2+}
b10	843.5055	y19	1783.7587
		y18	1712.7332
		y17	1641.6806
