# Supplementary material for: Thusin, a Novel Two-Component Lantibiotic with Potent Antimicrobial Activity against Several Gram-Positive Pathogens
Source: Front Microbiol. 2016 Jul 19;7:1115. doi: 10.3389/fmicb.2016.01115 (PMC4949975; doi:10.3389/fmicb.2016.01115)
Supplement: Supplementary file 5 [file Table3.PDF]

Table S3 Detailed MS/MS data of Thsβ'

Fragment ion	Measured *m/z*	Fragment ion	Measured *m/z*
b3	238.1186	y27	1244.6023^{2+}
a4	282.1815	y26	1203.0844^{2+}
b4	309.1558	y25	1161.5654^{2+}
b5	422.2398	y24	1126.0469^{2+}
b6	505.2765	y23	1069.5063^{2+}
b7	588.3147	y22	1033.9867^{2+}
b8	659.3526	y21	1995.9243
b9	772.4353	y20	1881.8401, 941.4237^{2+}
b10	843.5052	y19	1768.7542
		y17	1641.6812
